# Neighborhood risk factors for sports and recreational injuries: a systematic review of studies applying multilevel modeling techniques

**DOI:** 10.1186/s40621-022-00370-0

**Published:** 2022-02-21

**Authors:** Oluwatosin Ogunmayowa, Charlotte Baker

**Affiliations:** Department of Population Health Sciences, Virginia-Maryland College of Veterinary Medicine, Virginia Polytechnic Institute and State University, Blacksburg, VA 24061 USA

**Keywords:** Sports, Recreation, Injury, Neighborhood, Systematic review, Multilevel models

## Abstract

**Background:**

Sports and recreational activities are the most commonly reported cause of injury-related emergency department (ED) visits among children and young adults in developed countries, yet studies about the effect of neighborhood environment on sports and recreational injuries (SRI) are very limited. The aim of this study was to systematically review studies that apply multilevel modeling approach in examining the relationships between SRI and neighborhood-level risk factors.

**Data sources:**

A systematic search of peer reviewed English language articles was conducted in four electronic databases including PubMed (1992–2020), CINAHL (2000–2020), Sports Medicine and Education Index (1996–2020), and Web of Science (1991–2020).

**Study selection:**

Selected studies were observational or experimental studies of people of all ages across the world that assessed neighborhood risk factors for SRI (or all injuries including SRI) using multilevel regression analysis.

**Data synthesis:**

Nine studies—five cross-sectional, two prospective cohort, and two incidence studies—were selected out of a potential 1510. Six studies used secondary data and three used primary data. Only three studies examined SRI as the main or one of the main outcomes. These studies showed that neighborhood-level factors, such as higher socioeconomic context, lower street connectivity, and living or attending schools in urban communities, were associated with increased risk of SRI. Most studies did not provide a justification for the use of multilevel regression and the multilevel analytical procedure employed and quantities reported varied. The Quality Assessment Tool for Observational Cohort and Cross-Sectional Studies (National Institutes of Health) was used to assess the quality or risk of bias of each study. Four quality assessment criteria out of 15 were met by all nine studies. The quality assessment ratings of the reviewed studies were not correlated with the quality of information reported for the multilevel models.

**Conclusion:**

Findings from this review provide evidence that neighborhood-level factors, in addition to individual-level factors, should be taken into consideration when developing public health policies for injury prevention. Considering the limited numbers of studies that were identified by this systematic review, more multilevel studies are needed to strengthen this evidence in order to better inform SRI prevention policy decisions.

## Background

Exercise is now recognized as an important factor associated with improved health outcomes in people (Shephard 2003; Marshall and Guskiewicz 2003). It is reported to lower the risk of obesity, diabetes, cardiovascular disease, depression, anxiety and some cancer (Benefits of Physical Activity|Physical Activity|CDC 2021), and as a result, has been extensively promoted as part of a healthy lifestyle (Marshall and Guskiewicz 2003). Recent report shows increasing participation in physical activity in the USA with about 230 million Americans aged 6 years and over (76%) taking part in physical activities in 2020, up from 214 million in 2015 (73%) (Home|Pac Report 2021). While increased participation in physical activity provides many health benefits, increased exposure to physical activity also increases injury risk, posing a growing public health concern (Rui et al. 2019; Sheu et al. 2016).

Sports and recreational activities are the most commonly reported cause of injury-related emergency department (**ED**) visits among children and young adults in developed countries (Rui et al. 2019; Sheu et al. 2016). In the USA for instance, the average annual number of sports and recreational injuries (**SRI**) episodes is estimated to be about 8.6 million with more than 3 million resulting in visits to hospital ED (Sheu et al. 2016). This makes injury, including SRI, one of the main cause of morbidity, disability, and surplus health expenditures in children and young adults in developed countries (Haynes et al. 2003).

Although SRI poses a growing and important public health concern, studies about these injuries are limited compared to other types of injuries. One of the reasons for the poor attention to SRI problem is because they are often less severe than other types of injuries such as those from motor vehicle accidents and as a result, most injury surveillance systems for fatal and hospitalized injuries have excluded much of the burden of SRI with the exception of traumatic brain injury (**TBI**) (Marshall and Guskiewicz 2003; National Center for Catastrophic Sport Injury Research 2022). SRI are often included in state and national databases such as those from the Agency for Healthcare Research and Quality’s Health Care Utilization Project (Healthcare Cost and Utilization Project (HCUP)|Agency for Healthcare Research and Quality 2022) albeit without many injury-specific details such as playing surface. Additional SRI-specific surveillance exists for professional sports (Sprouse et al. 2020), collegiate sports (NCAA Injury Surveillance Program 2022), and active people of various ages (Goldberg et al. 2007; Gerson and Stevens 2004; National Electronic Injury Surveillance System All Injury Program 2019), yet are not inclusive of all physical activity-related injuries across the lifespan especially because many SRI do not require medical attention. To address this growing public health challenge, it is important to adequately document injuries resulting from sports and recreational activities and understand the socio-ecological factors (including individual and contextual) that influence SRI.

To date, only a few studies have examined how both individual (e.g., age, sex, or race/ethnicity) and contextual factors (e.g., neighborhood socioeconomic or built environments) are associated with SRI (Gropp et al. 2012; Mecredy et al. 2012; Simpson et al. 2005). The majority of studies focus on the individual-level factors while paying less attention to important contextual factors such as the neighborhood-built and socioeconomic environment. Research for bicycle or walking injuries related to motor vehicle crashes often involves analyzing geospatial data for contextual factors (such as street connectivity, access to sidewalks, bicycle lanes, parks, and recreational facilities.), yet this type of analysis is not frequently applied to the study of SRI. This limitation may be more closely related to the availability of contextual factors within SRI data sources, requiring more complex methods such as data linkage and analysis of nested or hierarchical data. This complexity is evident in the simple, and sometimes inappropriate, methods employed in studies assessing the influence of contextual factors on SRI risk. In studies where these associations are considered, the interactive effects between individual and contextual factors (cross-level interactions, **CLI**) are often not explored. The presence of CLI could cause variation in SRI risk among people with, for example, similar socioeconomic status but who live in neighborhoods with different access to parks and recreational facilities. Understanding CLI is important because it will help in understanding how individuals in characteristically different neighborhoods respond to interventions targeted at preventing or reducing the risk of SRI.

Classical regression approaches for analyzing the association between SRI and various individual and contextual factors are limited in their ability to simultaneously assess relationships occurring at multiple levels (Ogunmayowa 2020). This deficiency of the classical regression approach can be resolved by utilizing a multilevel modeling (**MLM**) approach for analyzing data that are nested or hierarchical in nature (Ogunmayowa 2020). Multilevel regression is an advanced form of classical regression that is appropriate for quantifying associations of hierarchically structured data (e.g., individual nested within neighborhood), as it can characterize associations within and between groups, and account for variation in outcome variables attributed to individual-level and neighborhood-level exposures (Woltman et al. 2012).

Despite the advantages MLM have over the classical regression approach, no systematic review has documented the application of this approach for analyzing the association between neighborhood risk factors and SRI. This review was carried out in order to encourage use of the multilevel approach in analyzing contextual data and to promote multilevel interventions in reducing SRI risks. A previous review examined how unintentional injury in childhood is related to neighborhood risk factors (McClure et al. 2015), while another review examined how fatal and non-fatal injuries are related to neighborhood socioeconomic factors (Ferdinand et al. 2012). In our review, we were interested in all neighborhood determinants of SRI alone in all age groups.

The main objective of this review is to systematically review studies that apply a multilevel modeling approach in assessing the relationships between SRI and neighborhood-level risk factors. The specific objectives are to: (1) examine how neighborhood-level risk factors is related with SRI when considered simultaneously with individual-level factors that influence SRI; (2) identify and characterize the multilevel methods or approach from articles selected for review; and (3) make recommendations on how to overcome identified gaps in research and statistical methodology. This study offers a valuable synthesis for policymakers, public health experts and other stakeholders concerned about reducing the burden of SRI.

## Main text

### Methods

#### Registration

This systematic review was designed following the guidelines of the Preferred Reporting Items for Systematic reviews and Meta-Analyses (**PRISMA**), and the protocol was registered with the International Prospective Register of Systematic Reviews (**PROSPERO**) on January 18, 2021 (Registration Number: CRD42021227119).

#### Eligibility criteria

Studies were selected for our systematic review based on the following criteria: (1) Study types: we included observational studies such as case–control studies, cohort studies, incidence studies, prevalence studies, cross-sectional studies, and longitudinal studies. We also included experimental studies such as randomized controlled trials. Studies were restricted to journal articles reported in English language with review articles and meta-analysis excluded from our study. (2) Participants: we included studies that examined human population of all age groups in countries across the world. (3) Exposures: we included studies that examined neighborhood-level exposure variables that are risk factors for SRI, such as built or physical environment, neighborhood socioeconomic environment, neighborhood social vulnerability, neighborhood social inequality, and neighborhood social capital, in addition to individual-level risk factors. Our definition of socioeconomic environment did not include social capital. Socioeconomic environment was defined as the intersection of social and economic factors that determine the distribution of resources, money and power in a community (Lantz and Pritchard 2010; Socioeconomic Environment—The Collaborative on Health and the Environment 2022). This is often determined by social standing factors such as marital status, occupation, religion, family, income, class, or age (Socioeconomic Environment—The Collaborative on Health and the Environment 2022) and is related to the strength of your social cohesion (i.e., social relationships) (Social Cohesion|Healthy People 2020). The stronger your social cohesion, the more likely you are to be able to rely on others to help you when you need it and the greater your social capital. Social capital was defined as the network you belong to and the types of values you hold (Social Cohesion|Healthy People 2020). Your social capital may influence your socioeconomic environment, but this is tempered by socioeconomic determinants. (4) Outcomes: we included studies in which the outcome (or one of the outcomes) was SRI; outcome included a broader injury category while exposure variables in the multilevel model included sports and recreation activities, playgrounds and recreational facilities, or other sports and recreation-related exposure variables; or outcome included a broader injury category while sports and recreational activities and/or playgrounds and recreational facilities were reported as one of or the main risk factors for injury. SRI was defined as damage to the body caused by exposure to an external force related to sport, recreation, or physical activity. (5) Data analysis: we included studies that used multilevel regression analysis to examine the association between individual-level and neighborhood-level exposures and SRI (or all injuries including SRI).

#### Information sources

The search for studies that meet our eligibility criteria was conducted in four electronic databases which include: (1) PubMed (1992–Present), (2) CINAHL from EBSCOhost (2000–Present), (3) Sports Medicine and Education Index (Proquest) (1996–Present), and (4) Web of Science from Clarivate Analytics (1991–Present). Reference lists of previously published systematic reviews were also scanned for additional studies. The final search of electronic databases was run on December 3, 2020. The same search term was used in all the electronic databases; however, the search filters varied depending on the options available in each database.

#### Search strategy

We conducted literature searches for systematic reviews related to our topic in several electronic databases and PROSPERO to ensure that no previous or ongoing studies has been or was being carried out on our planned topic of study. We then developed our search strategy in consultation with a librarian with expertise in systematic reviews in Population Health Sciences. Based on the eligibility criteria listed above, we developed search terms that covered a wide range of articles related to our topic using special symbols, including truncation and quotation marks, and Boolean operators, to combine search words or phrases. Literature search words or phrases were developed using a combination of test words or phrases related to sports and recreational activities, injuries, contextual exposure variables, and multilevel modeling. We used the same search terms in all the electronic databases and limited our literature search to human subjects, English language, and peer review academic journals. The search strategy used in the electronic databases is listed below:

(environment* OR context* OR “built environment” OR “physical environment” OR neighborhood OR neighbourhood OR “neighborhood environment” OR “neighbourhood environment” OR “neighborhood built environment” OR “neighbourhood built environment” OR “neighborhood physical environment” OR “neighbourhood physical environment” OR communit* OR municipal OR urban* OR city OR cities OR town OR towns OR walkability OR connectivity OR built OR building* OR street OR streets OR “green space” OR greenspace OR park OR “recreation* facilit*” OR “environmental design” OR “socioeconomic” OR “socioeconomic status” OR “neighborhood socioeconomic status” OR “socioeconomic environment” OR “social environment” OR “social inequit*” OR “social inequalit*” OR “social determinant*” OR “political system*” OR “health disparit*” OR “social identification” OR “racial composition” OR “social vulnerability index” OR “residence characteristics” OR “residential segregation” OR “income inequit*” OR “income inequalit*”) AND (“motor activit*” OR sport OR sports OR athlete* OR recreation* OR “leisure activit*” OR “physical fitness” OR “physical exertion” OR “physical endurance” OR “physical activit*” OR exercis* OR “active living” OR “active lifestyle*” OR play OR “outdoor activit*” OR walk* OR run OR running OR bike OR biking OR bicycle OR bicycling OR cycle OR cycling OR “active transport*” OR “active transit” OR “active commuting” OR “physically active” OR fitness OR baseball OR basketball OR boxing OR “cricket sport” OR football OR golf OR gymnastics OR hockey OR “martial arts” OR mountaineering OR “racquet sports” OR tennis OR jog OR jogging OR skating OR “snow sport*” OR skiing OR soccer OR “track and field” OR volleyball OR walking OR “water sports” OR swimming OR “weight lifting” OR wrestling OR camping OR dancing OR hobbies OR gardening) AND (injury OR injuries OR wound OR wounds OR trauma* OR rupture OR fracture OR sprain* OR strain* OR avulsion OR concussion) AND ("multilevel model*" OR "multi-level model*" OR "multilevel regression" OR "multi-level regression" OR "multilevel analysis" OR "multi-level analysis" OR “multilevel logistic regression” OR “multi-level logistic regression” OR “hierarchical model*” OR “hierarchical regression” OR “hierarchical linear model” OR “hierarchical logistic regression” OR “random effects model*” OR “random coefficient model*” OR “mixed model*” OR “mixed effect model*”) NOT ("systematic review" OR “systematic analysis” OR "literature review" OR review OR “meta-analysis”).

#### Data management

Literature search results from the four electronic databases were uploaded into a citation manager, EndNote. Literature search results were then exported from EndNote into COVIDENCE, a web-based software that facilitates collaboration between reviewers during the process of screening and selection of articles. Duplicate articles were automatically removed by COVIDENCE, and proper verification of articles’ details was followed to ensure that they were actual duplicates.

#### Selection process

Screening of titles and abstracts of articles generated from electronic databases search results was carried out by the two authors using COVIDENCE software. The screening was based on the eligibility criteria for articles listed previously. Articles that appeared to meet our inclusion criteria were selected and went through full text screening by both reviewers. Disagreements between the two authors were resolved by discussion.

#### Data collection process

Articles selected after full text screening went through the data extraction stage which was carried out by the first author and verified by the second author. Data extraction sheet was developed following the Matrix Method (Garrard 2017), and the following information was collected: name of first author, year of publication, title of article, name of publication journal, country of study, study design, participant information (e.g., sample size, age, sex), data type and date of collection, geographical extent of neighborhood, neighborhood-level measures, individual-level measures, measures of outcomes (i.e., SRI or total injuries including SRI), statistical method used, and main findings.

#### Study quality assessment

The quality assessment tool for observational cohort and cross-sectional studies of the National Institute of Health (NIH) was used to assess the quality or risk of bias of each study selected in our systematic review (Study Quality Assessment Tools|NHLBI, NIH 2021). The NIH tool is based on 14 assessment criteria with a score of yes, no, cannot determine (CD), not applicable (NA), and not reported (NR) for each of the criteria. In addition to the NIH 14 criteria, an assessment criterion that determined if enough information was provided to know if the appropriate multilevel approach had been used was included. Therefore, a total number of 15 points were awarded to studies based on the number of “yes” answers and each study’s quality and risk of bias were assessed on items related to research question, study population, sample size justification, exposure and outcome measurements, participation and follow-up rates, and statistical analyses. The quality assessment of studies was independently conducted by the two authors. Discrepancies were resolved by discussion.

### Results

**Fig. 1:**
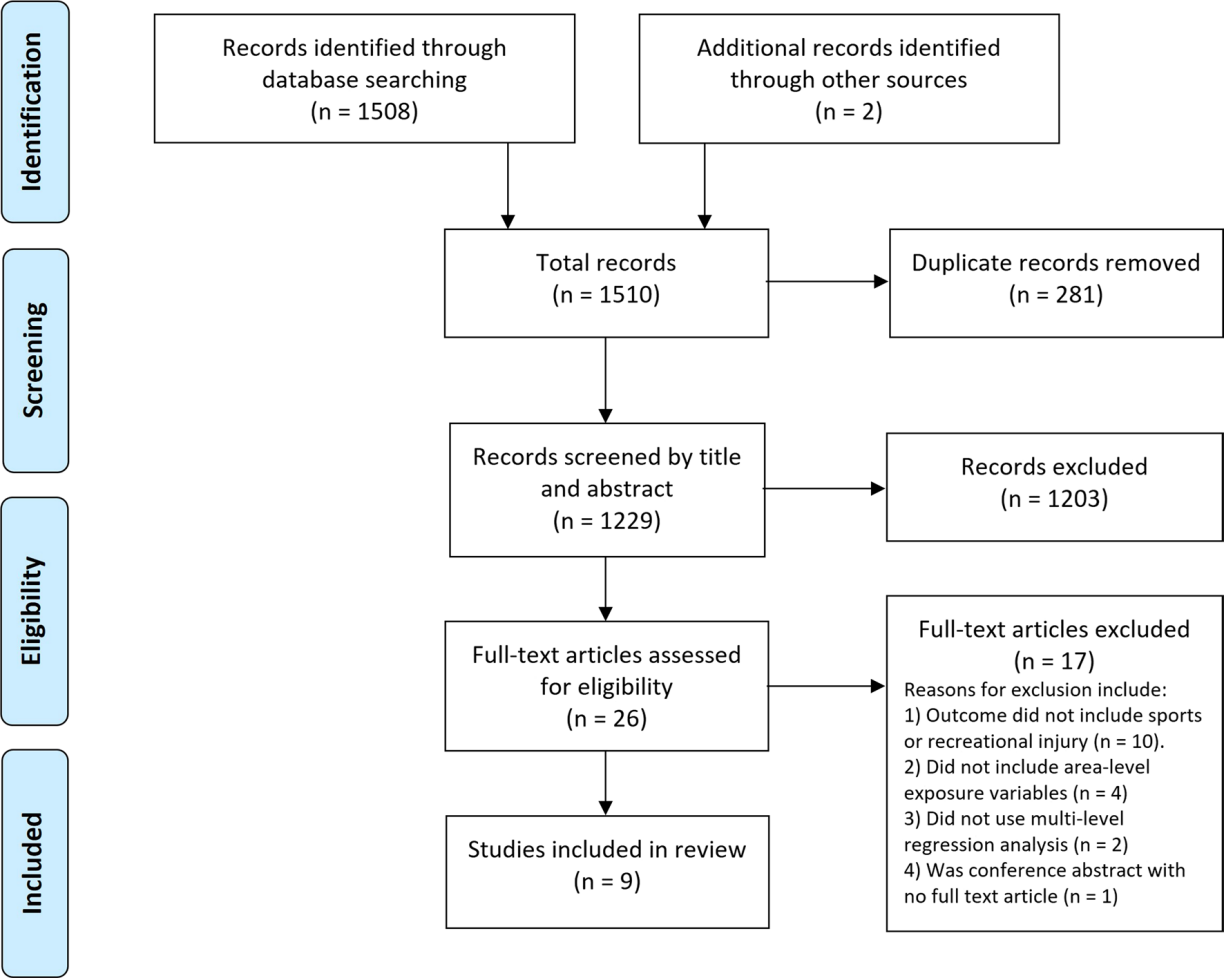
PRISMA flowchart of study selection

Of the 1510 records identified from searching four electronic databases and reference lists of other studies, only nine (9) were included in our systematic review analysis (Fig. 1).

#### Study characteristics

The characteristics of the studies included in this systematic review are summarized in Table 1. Among the final nine studies selected for the systematic review, five were cross-sectional studies (Gropp et al. 2012; Mecredy et al. 2012; Simpson et al. 2005; Pattussi et al. 2006; Byrnes et al. 2015), two were prospective cohort studies (Kendrick et al. 2005; Mutto et al. 2012), and another two were incidence studies (Haynes et al. 2003; Sellström et al. 2003) [Pearce 2012]. Six of the nine studies used secondary data collected by other organizations or from records of emergency department visits (Haynes et al. 2003; Gropp et al. 2012; Mecredy et al. 2012; Simpson et al. 2005; Byrnes et al. 2015; Sellström et al. 2003) while the remaining three used primary data collected by the researchers directly (Pattussi et al. 2006; Kendrick et al. 2005; Mutto et al. 2012). Almost half of the studies (four) were carried out in North America (all four of them in Canada and, surprisingly, none in the USA) (Gropp et al. 2012; Mecredy et al. 2012; Simpson et al. 2005; Byrnes et al. 2015), followed by Europe (three in total; two in the United Kingdom (Haynes et al. 2003; Kendrick et al. 2005) and one in Sweden (Sellström et al. 2003)), South America [one in Brazil (Pattussi et al. 2006)], and Africa [one in Uganda (Mutto et al. 2012)].

**Table 1:** Characteristics of studies included in the review

Study ID	Authors and year of publication	Title and journal	Purpose of study	City/ Region/ Country	Study design	Study population/ number of data level	Age (year)	Male (%)	Type of injury data and date of collection	Geographical extent of neighborhood
1	Haynes, Reading & Gale. 2003	Household and neighborhood risks for injury to 5–14 year old children. *Soc Sci Med*	Are household and neighborhood risk factors independently associated with injury in children?	Norwich, United Kingdom	Incidence study	22,771 children in total. Three levels: individual/family, enumeration districts (*N* = 347) and social areas (*N* = 21)	5–14	51.2 days at risk	Secondary data; 1999/2000 accident records of the Accident and Emergency Department at Norfolk/Norwich Hospital	Enumeration districts (which are composed of about 150–200 households and are the smallest area with available census data), and social areas (i.e., groups of neighboring enumeration districts with similar Townsend material deprivation index)
2	Sellström, Guldbrandsson, Bremberg, Hjern & Arnoldsson. 2003	Association between childhood community safety interventions and hospital injury records: a multilevel study. *J Epidemiol Community Health*	How does safety measures in overall municipal, preschool, school, and recreational activity settings affect the risk of admitting children and adolescents to hospital due to injury?	Stockholm County, Sweden	Incidence study	1,056,064 person-years. Two levels: individual/family and community (*N* = 25)	1–15	NR	Secondary data; 1995–1999 children's injuries records obtained from the Hospital Discharge Register. Each child was followed for one year	Municipalities with each having an average population of 40,000 inhabitants. Stockholm city was excluded because of its large population size
3	Kendrick, Mulvaney, Burton, & Watson. 2005	Relationships between child, family and neighborhood characteristics and childhood injury: A cohort study. *Soc Sci Med*	How are child, family, and neighborhood characteristics associated with childhood unintentional injuries that were medically attended to?	Nottingham, United Kingdom	Prospective cohort study	2357 children. Three levels: individual, family (*N* = 1717) and electoral wards (*N* = 70)	0–7	52.3	Primary data gathered from a cohort study that was nested in a randomized controlled trial's control arm of primary care injury prevention in children	Electoral ward
4	Simpson, Janssen, Craig, & Pickett. 2005	Multilevel analysis of associations between socioeconomic status (SES) and injury among Canadian adolescents. *J Epidemiol Community Health*	How are individual- and neighborhood-level socioeconomic variables associated with the occurrence of medically-treated, hospitalized, fighting, and sports/recreational injuries among Canadian adolescents?	Canada	Cross-sectional study	7235 students. Two levels: individual/family and school's neighborhood (*N* = 170)	11–16 (Grade 6–10)	46.4	Secondary data; 2001/2002 Health Behavior in School-age Children survey	5 km buffer around each school attended by students who responded to the survey
5	Pattussi, Hardy, & Sheiham. 2006	Neighborhood social capital and dental injuries in Brazilian adolescents. *Am J Public Health*	How is neighborhood social capital associated with dental injury?	Cities of Taguatinga and Ceilândia of Distrito Federal, Brazil	Cross-sectional study	1302 adolescents: Two levels: individual/family and school's neighborhood (*N* = 37)	14–15	52.3	Primary data from clinical examination and self-administered questionnaire in 2002	Enumeration districts aggregates composed of an average of about 3,535 and 13,158 households and individuals, respectively

Study ID	Authors and year of publication	Title and journal	Purpose of study	Country	Study design	Study population/ number of data level	Age (year)	Male (%)	Type of injury data and date of collection	Geographical extent of neighborhood
6	Mecredy, Janssen, & Pickett. 2012	Neighborhood street connectivity and injury in youth: a national study of built environments in Canada. *Inj Prev*	How is street connectivity associated with injuries among Canadian youths?	Canada	Cross-sectional study	9021 students: Two levels: individual/family and school's neighborhood (*N* = 180)	11–15 (Grade 6–10)	47.5	Secondary data; 2006 Health Behavior in School-age Children survey	5 km circular buffer around each school attended by students who responded to the survey
7	Mutto, Lawoko, Ovuga, & Svanstrom. 2012	Childhood and adolescent injuries in elementary schools in north-western Uganda: extent, risk and associated factors. *Int J Inj Contr Saf Promot*	What are the extent, nature and risk factors of childhood injuries in elementary schools in north-western Uganda?	North-western Uganda	Prospective cohort study	1000 students. Two levels: individual and school (*N* = 13)	9–16 (Grade 5)	54.5	Primary data from injury register completed by Grade-5 teachers in one school term that lasted from February 2 to April 30, 2009	Not specified
8	Gropp, Janssen & Pickett. 2012	Active transportation to school in Canadian youth: should injury be a concern? *Inj Prev*	How is active transportation to school associated with injury in Canadian youth?	Canada	Cross-sectional study	20,076 students in total. Two levels: individual/family and school's neighborhood (*N* = 419)	11–15	36.4	Secondary data; 2009/2010 Health Behavior in School-age Children survey	1 km buffer around each school attended by students who responded to the survey
9	Byrnes, King, Hawe, Peters, Pickett & Davison. 2015	Patterns of youth injury: a comparison across the northern territories and other parts of Canada. *Int J Circumpolar Health*	How does injury occurrence and its potential risk factors among youths in the northern territories of Canada compare with other parts?	Canada	Cross-sectional study	26,078 students in total. 3942 students attended 80 schools located in the northern territories. Two levels: individual/family and school's neighborhood (*N* = 80)	11–15	49	Secondary data; 2009/2010 Health Behavior in School-age Children survey	1 km buffer around each school attended by students who responded to the survey

The size of the study population at the individual level ranged from 1000 participants to 1,056,064 person-years (Median = 9021) with four of the studies having a population greater than 20,000. All nine articles included in our review studied children and adolescent (≤ 16 years old) even though all age groups were considered in this study. For studies in which the sex of participants was reported, approximately half of the populations were male.

In seven studies, individual and family were combined into a single exposure level (individual/family), resulting in two exposure levels for analysis—individual/family and neighborhood. In another study, individual and family were considered as distinct exposure levels, resulting in a total of three exposure levels—individual, family, and neighborhood (Kendrick et al. 2005). In the final study, neighborhood was divided based on size resulting in three exposure levels under consideration—individual, enumeration district (i.e., smallest area with available census data), and social area (i.e., a group of enumeration districts) (Haynes et al. 2003). About half of all studies (4) defined the geographical extent of neighborhood by administrative area (e.g., municipality) and census area (i.e., geographical area defined for counting and recording information about a population) (Haynes et al. 2003; Pattussi et al. 2006; Kendrick et al. 2005; Sellström et al. 2003), while another 4 defined neighborhood by buffers (i.e., a specified distance surrounding a geographic feature such as school) (Gropp et al. 2012; Mecredy et al. 2012; Simpson et al. 2005; Byrnes et al. 2015). For studies that used buffers to define neighborhood, a 1 km or 5 km radius was used as buffer size.

Neighborhood-level exposure variables used in all studies can be grouped into five main categories (Table 2), and they included: socioeconomic environment, physical environment, neighborhood crime levels and safety measures, social capital and social cohesion, and urban–rural geographic location; with socioeconomic environment being the most commonly studied neighborhood-level variable (*n* = 7 studies). Examples of socioeconomic neighborhood environment variables considered include: material deprivation, home ownership status, immigration status, lone parent status, poverty, housing value, employment status, education, and income while the examples of physical environment variables considered include: playgrounds, parks and recreational facilities, population density, street or road connectivity, permanent road access, pedestrian controlled lights, zebra crossings, total road lengths, etc.

**Table 2:** Individual and contextual measures, and main study outcomes

Study ID	Authors and year of publication	Neighborhood-level measures	Individual/family-level measures	Main outcome measures for sports and recreational (**SRI**) injuries or total injuries including SRI
1	Haynes, Reading & Gale. 2003	Townsend material deprivation score, accommodation renters (%), pre-school (0–4 years) accident/injury rate, distance to playground, distance to hospital, migrants (%), 5–14 years old (%), and social cohesions indicators including: people who changed home in the past year (%), lone parents household (%), single persons household (%)	Age, sex, number of children, number of adults, and age difference between children and the oldest woman in the household	Total injuries and serious injuries (for a 13-month period). 25% of injuries were sports related. Although the proportion of SRI were not explicitly reported, 15% of injuries were reported to have occurred at a sports/recreational facility, playground or park
2	Sellström, Guldbrandsson, Bremberg, Hjern & Arnoldsson. 2003	Safety index, and population density	Age, sex, maternal education, maternal birth's country, and social allowance	Injuries that fall between E830–E929 in ICD-9 or W 01–X 59 in ICD-10 (for a 12-month period). Transportation-related injuries were excluded, and data was limited to one injury per person-year. The proportion of injuries that was due to sports and recreation activities were not reported; however, the level of safety measures in recreation activity settings were reported as one of the neighborhood-level exposure variables
3	Kendrick, Mulvaney, Burton, & Watson. 2005	Child poverty index, geographical access to services; distance from hospital; crime reported to police (%): dwellings experiencing domestic burglaries, population experiencing vehicle crime, population experiencing violent crime, and housing of lowest value; facilities (number/1000 children < 5 years old): nursery places, child minder places, parks and play areas, and leisure centers; road safety measures (number/1000 children < 5 years old): school crossing patrols, zebra crossings, pedestrian controlled lights, small areas of traffic calming, and large areas of traffic calming	(1) Child-level characteristics: Age, and sex. (2) Family-level characteristics: teenage motherhood; 4 or more children under age 16 in family; single parent family; rented accommodation; number of unemployed parents; car ownership; receives means tested benefits; and safety practices: fitted stair gates, fitted and working smoke alarms, and safe storage of sharp objects in kitchen	Primary care attendance, accident and emergency department attendance, and hospital admission rates for unintentional injuries (for a two-year follow-up period)
4	Simpson, Janssen, Craig, & Pickett. 2005	Lone parent families (%), unemployment (%), residents with less high school education (%), and average employment income	Age, sex, family affluence, poverty, local area safety's perception, residential area's perception, and perceived family wealth	Any medically-treated injury, hospitalized injury, sports/recreational injury, and fighting injury (for 12 months preceding the survey). 58.4% of medically treated injury were sports/recreation-related
5	Pattussi, Hardy, & Sheiham. 2006	Social capital, infrastructure, and poverty gap	Age, incisal overjet, lip coverage, body mass index (BMI), and social class	Prevalence of dental injuries in boys and girls. Sports were reported to cause 13.3% of the dental injuries while playing caused 48.1% of the dental injuries

Study ID	Authors and year of publication	Neighborhood-level measures	Individual/family-level measures	Main outcome measures for sports and recreation- related (SR) injuries or total injuries including SR injuries
6	Mecredy, Janssen, & Pickett. 2012	Street connectivity, socioeconomic status, urban–rural geographic location, and parks/recreational facilities	Gender, grade, family affluence, perceived neighborhood safety, and perceived neighborhood aesthetics	Any medically-treated street injury (for 12 months preceding the survey). Street injury includes those that occurred in the street/road/parking lot and during a physical activity
7	Mutto, Lawoko, Ovuga, & Svanstrom. 2012	School, urban–rural location, schools' religious affiliation	Age, and sex	School-related injuries. 35% of injuries occurred on playgrounds. 32.5% of injuries occurred during sporting activities while 12.5% occurred while walking
8	Gropp, Janssen & Pickett. 2012	Urban/rural geographic status, average precipitation levels, total road lengths, street or road connectivity, speed limits within 1 km buffer of each school, and population estimates of median household income for 2006	Age, gender, ethnicity, perceived family socioeconomic status, perceived residential neighborhood safety and participation in organized sports	Active transportation injuries (which includes both walking/running and bicycling injuries), walking/running injuries, and bicycling injuries (for a 12-month period)
9	Byrnes, King, Hawe, Peters, Pickett & Davison. 2015	Population size, Aboriginal composition (%), permanent road access, Dwellings requiring major repair (%), alcohol policy in 2008	Sex, school grade, ethnicity, relative family affluence, impaired driving as passenger or driver in the past 30 days, helmet's use when riding motorized vehicles in the last 12 months, alcohol use, organized sports participation	Any injury (for a 12-month period). 23.5% of injuries among northern youths were SRI. Only walking/running injury was reported for recreation-related injuries. 11.9% of injuries in northern territories occurred at a sports facility/field

Only three of the studies considered SRI as an outcome or one of the outcome variables (Table 2) (Gropp et al. 2012; Mecredy et al. 2012; Simpson et al. 2005). The other studies focused on a broader category of injury as outcome variable but included sports and recreation-related exposure variables in the multilevel model or reported sports and recreation-related activities or facilities as a main cause of injury.

#### Study quality assessment

The report for the quality assessment of studies included in this systematic review is contained in Table 3. Four quality assessment criteria were met by all studies, and they include: (1) clearly specified and defined study population, (2) study subjects’ selection from similar population at same time period, and pre-specification and uniform application of inclusion and exclusion criteria for all study participants, (3) statistical adjustment for key confounding variables, and (4) provision of adequate information to know that the appropriate multilevel technique was used and applied correctly. Where applicable, all studies measured different level for exposures that can vary in amount or level, and all studies, except one, provided clearly stated research objectives. Justification of sample size, description of statistical power, or estimation of variance and effect size were provided in six of the studies. Only a few studies provided report of study participation rate of ≥ 50% for eligible persons (*n* = 4), measured exposures of interest prior to measuring study outcomes (*n* = 2), and had sufficient timeline to increase the probability of finding significant association between exposure and outcome if it existed (*n* = 2). The median assessment score for included studies is 7 out of 15 (range 5–11).

**Table 3:** Quality assessment tool for observational cohort and cross-sectional studies by the National Institute of Health (NIH)

Criteria		Study ID								
		1	2	3	4	5	6	7	8	9
1	Was the research question or objective in this paper clearly stated?	No	Yes	Yes	Yes	Yes	Yes	Yes	Yes	Yes
2	Was the study population clearly specified and defined?	Yes	Yes	Yes	Yes	Yes	Yes	Yes	Yes	Yes
3	Was the participation rate of eligible persons at least 50%?	NR	Yes	Yes	NR	Yes	Yes	NR	NR	NR
4	Were all the subjects selected or recruited from the same or similar populations (including the same time period)? Were inclusion and exclusion criteria for being in the study prespecified and applied uniformly to all participants?	Yes	Yes	Yes	Yes	Yes	Yes	Yes	Yes	Yes
5	Was a sample size justification, power description, or variance and effect estimates provided?	Yes	Yes	Yes	No	Yes	No	Yes	Yes	No
6	For the analyses in this paper, were the exposure(s) of interest measured prior to the outcome(s) being measured?	No	No	Yes	No	No	No	Yes	No	No
7	Was the timeframe sufficient so that one could reasonably expect to see an association between exposure and outcome if it existed?	No	No	Yes	No	No	No	Yes	No	No
8	For exposures that can vary in amount or level, did the study examine different levels of the exposure as related to the outcome (e.g., categories of exposure, or exposure measured as continuous variable)?	Yes	Yes	Yes	Yes	Yes	Yes	Yes	Yes	NA
9	Were the exposure measures (independent variables) clearly defined, valid, reliable, and implemented consistently across all study participants?	No	No	No	No	No	No	No	No	No
10	Was the exposure(s) assessed more than once over time?	NA	NA	NA	NA	NA	NA	NA	NA	NA
11	Were the outcome measures (dependent variables) clearly defined, valid, reliable, and implemented consistently across all study participants?	Yes	Yes	Yes	No	Yes	No	No	No	No
12	Were the outcome assessors blinded to the exposure status of participants?	NA	NA	NA	NA	NA	NA	NA	NA	NA
13	Was loss to follow-up after baseline 20% or less?	NA	NA	NR	NA	NA	NA	Yes	NA	NA
14	Were key potential confounding variables measured and adjusted statistically for their impact on the relationship between exposure(s) and outcome(s)?	Yes	Yes	Yes	Yes	Yes	Yes	Yes	Yes	Yes
15	Was enough information provided to know that the appropriate multilevel technique/approach had been used and appropriately applied?	Yes	Yes	Yes	Yes	Yes	Yes	Yes	Yes	Yes
	**Total Score**	7	9	11	6	9	7	10	7	5

*CD cannot determine; NA, not applicable; NR, not reported

#### Multilevel analysis assessment

Among the nine studies included in our systematic review, only three assessed and reported the variance in SRI or any injuries due to neighborhood-level differences for the null or unconditional model(s) (Haynes et al. 2003; Gropp et al. 2012; Mutto et al. 2012) (Table 4). Among the three that reported the variance in neighborhood-level differences, only two reported the statistical significance of the variance, the intraclass correlation coefficient (**ICC)** or the variance partition coefficient (**VPC**) in order to justify the use of multilevel analysis (Haynes et al. 2003; Gropp et al. 2012). Of the nine studies included in the review, only two evaluated and reported the variance in individual slopes (random effects) (Simpson et al. 2005; Kendrick et al. 2005) while another two tested for cross-level interactions between individual-level and neighborhood-level exposure variables in order to account for the individual slope variances where they existed (Pattussi et al. 2006; Sellström et al. 2003); however, these two studies failed to report if there was variance between individual slopes. Five studies of those included in the review assessed or reported about the unexplained variance at neighborhood-level or the proportion of variance explained by the neighborhood-level variables for the final multilevel model(s) (Haynes et al. 2003; Pattussi et al. 2006; Kendrick et al. 2005; Mutto et al. 2012; Sellström et al. 2003).

**Table 4:** Summary of statistical approach of included studies

Study ID	Authors and year of publication	Was variance in SRI (or any injury) due to neighborhood-level differences assessed or reported for unconditional or null model(s) to justify the use of multilevel model?	Was variance in individual slopes (random effects) evaluated or reported and was cross-level interaction tested for to account for the variance where it existed?	Was the unexplained variance at neighborhood-level or the proportion of variance explained by the neighborhood-level variables assessed or reported for the final multilevel model(s)?	Model-building process employed to develop final model(s)	Multilevel modeling type and software used
1	Haynes, Reading, Gale. 2003	Yes. Unexplained variance for the null or unconditional models were reported and tested for significance; however, ICCs were not reported	No	Yes. The unexplained variances at both the enumeration district and social area levels were reported	A single multilevel model was developed; however, several variations of multilevel models (including null model, single level, and different combinations of two- and three-level models) were developed to assess the unexplained variance in models	Multilevel logistic regression using MLwiN software package
2	Sellström, Guldbrandsson, Bremberg, Hjern & Arnoldsson. 2003	No	No, variance in individual slopes was not evaluated or reported. But cross-level interactions were tested for. Interaction terms were, however, excluded from final models because they were not statistically significant	Yes	Three multilevel models of increasing complexities were developed to adjust for potential confounders	Multilevel logistic regression using SAS makro Glimmix software package
3	Kendrick, Mulvaney, Burton, & Watson. 2005	No	Yes. Two-way interactions were examined where it appeared they might exist; however, it is not stated if the interactions included cross-level interactions	Yes	A forward selection approach was used	Multilevel Poisson regression using MLwiN software package
4	Simpson, Janssen, Craig, & Pickett. 2005	No	Yes, it was reported that there was no significant variation in the slopes of the relationship between each socioeconomic variables and medically-treated injury across neighborhoods. For other injury outcomes, the variation in slopes were therefore assumed to be non-significant	No	A two-step process was employed which include: (1) fitting bivariate models, and (2) fitting multivariable multilevel models from significant socioeconomic exposure variables in the bivariate models. Age and sex were included in all multilevel models	Multilevel logistic regression using MLwiN software package
5	Pattussi, Hardy, & Sheiham. 2006	Not clear. It appears that a null model may have been fitted based on descriptive results of between-neighborhood variation in dental injuries; however, this was not clearly reported	No, variance in individual slopes was not reported. However, results of the sex-stratified multilevel models mean that cross-level interactions were tested for between sex and neighborhood-level variables	Yes, the variance explained by neighborhood-level variable was assessed based on the report that most of the between-neighborhood variation in dental injuries was explained by social capital; however, no numerical value of the proportion of variance explained was reported	Four series of pre-determined multilevel models were developed including final models containing both individual and neighborhood-level predictor variables	Multilevel logistic regression using MLwiN software package
6	Mecredy, Janssen, & Pickett. 2012	No	No	No	Three series of pre-determined multilevel models were developed including a final model for total street injuries that contained both individual- and neighborhood-level predictors fitted using a backward elimination multilevel regression method. Predictor variables in the final reduced multilevel model were also then used to develop four physical activity-specific injury multilevel models	Multilevel logistic regression using SAS Glimmix procedure
7	Mutto, Lawoko, Ovuga, & Svanstrom. 2012	Variance due to neighborhood level was reported for the null model; however, statistical significance of the variance, and/or the variance partition coefficient (VPC) for the model was not reported to justify the use of a multilevel model	No	Yes, the neighborhood level variance and the proportional change in variance (PCV) explained by the final model was reported	Four series of pre-determined multilevel models were developed including a null model, and final model that included both individual and neighborhood-level predictor variables	Multilevel logistic regression using STATA
8	Gropp, Janssen & Pickett. 2012	Yes. Intraclass correlation coefficient (ICC) was reported	No	No	A backward elimination approach was used to select statistically significant individual and area-level predictor variables	Multilevel logistic regression using SAS software package
9	Byrnes, King, Hawe, Peters, Pickett & Davison. 2015	No	No	No	A backward elimination approach was used but sex, grade, and relative family affluence were retained in the final model based on a priori decision	Multilevel, multivariable, log binomial regression model was used to analyze risk of injury among northern youths only. Modeling was carried out with SAS software package

#### Key findings

The estimated effects of neighborhood-level factors on SRI (and additional injuries) are summarized in Table 5. Among the nine studies included in this systematic review, only three examined SRI as an outcome or one of the outcome variables (Gropp et al. 2012; Mecredy et al. 2012; Simpson et al. 2005), while the others focused on a broader category of injury as outcome variable with sports and recreational activities or facilities included in the multilevel model as an exposure variable or with sports and recreational activities or facilities reported as a main cause of injury.

**Table 5:** Statistically significant (*p* < 0.05) neighborhood-level effects in final multilevel models for included studies

Study ID	Authors and year of publication	Estimated effects of significant neighborhood-level variables on SRI or total injuries including SRI in final multilevel model(s)	Summary of study's main findings
1	Haynes, Reading, Gale. 2003	Social area material deprivation was positively correlated with the risk of all injuries and serious injuries. The risk of all injuries and serious injuries increased by 4% for each unit increase in Townsend material deprivation score (OR_all_ = 1.04, 95%CI = 1.02–1.06; OR_serious_ = 1.04, 95%CI = 1.02–1.07)	Neighborhood material deprivation increased the risk of all injuries and serious injuries
2	Sellström, Guldbrandsson, Bremberg, Hjern & Arnoldsson. 2003	Few and average level of safety measures (lower safety index) were positively associated with higher hospital admissions rate for injuries in preschool-aged children. The odds of being admitted for injuries were greater by 20% (RR_average_ = 1.20, 95%CI = 1.05–1.36) and 33% (RR_few_ = 1.33, 95%CI = 1.15–1.49) in municipalities with average and few safety measures, respectively, compared with those with many safety measures. In school-aged children, positive association was also observed between lower safety index and hospital admissions rate; however, the relationships were not statistically significant	Lower level of safety measures increased the risk of injuries for preschool-aged children
3	Kendrick, Mulvaney, Burton, & Watson. 2005	(1) Primary care attendance rates for injuries were 2.4 (RR = 2.41, 95%CI = 1.34–4.34) and 1.9 (RR = 1.92, 95%CI = 1.04–3.52) times greater in children living in the 3rd and 4th deprived quintile of wards per geographical access to services, respectively, than those living in the least deprived quintile of wards. However, the attendance rates for injuries were not significantly greater in children living in the 2nd (RR = 1.22, 95%CI = 0.65–2.29) and most deprived (5th) quintile (RR = 1.65, 95%CI = 0.90–3.03) of wards	(1) The relationship between neighborhood access to health care services and primary care attendance rates for injuries in children was n-shaped
		(2) Accident and Emergency Department attendance rate for injuries (in model that included rented accommodation) increased by 2% for each additional increase in the parks and play areas per 1000 children < age 5 (RR = 1.02, 95%CI = 1.00–1.04)	(2) The rates of visiting Accident and Emergency Departments for injuries increased with higher number of parks and play areas in wards
		(3) Hospital admission rates for injuries in model that included fitted stairgate were 5.2 (RR = 5.21, 95%CI = 1.52–17.90) and 4.5 (RR = 4.50, 95%CI = 1.32–15.40) times greater in children living in the 2nd and most deprived quarter of wards per child poverty index, respectively, than those living in the least deprived quarter of wards. Also, hospital admission rates for injuries in model that included smoke alarm were 7.0 (RR = 7.04, 95%CI = 2.07–23.94), 4.2 (OR = 4.23, 95%CI = 1.16–15.40) and 4.1 (RR = 4.13, 95%CI = 1.17–14.64) times greater in children living in the 2nd, 3rd, and most deprived quarter of wards per child poverty index, respectively, than those living in the least deprived quarter of wards. In addition, hospital admission rates for injuries in the fitted stairgate model increased by 14% (RR = 1.14, 95%CI = 1.03–1.27) for every percent increase in population experiencing violent crimes	(3) Hospital admission rates for injuries were higher in wards with higher child poverty index than those with lower index. Also, admission rates were higher in wards where a greater proportion of the population were experiencing violent crimes
4	Simpson, Janssen, Craig, & Pickett. 2005	(1) The odds of being hospitalized for injuries was 64% greater in schools' neighborhood with high (OR = 1.64, 95%CI = 1.04–2.61) and very high (OR = 1.64, 95%CI = 1.05–2.56) percentages of lone parent families compared with those with low percentage. Also, the odds of hospitalization for injuries was more than 2 times greater in schools' neighborhood with a very high percentage of population with less than a high school education compared with those with low percentage (OR = 2.11, 95%CI = 1.36–3.28)	Lower socioeconomic status increased the risk of injury hospitalization
		(2) The odds of having sports/recreational injury were 20% (OR = 0.80, 95%CI = 0.67–0.96) and 19% (OR = 0.81, 95%CI = 0.68–0.97) lower in schools' neighborhood with medium and high average employment income, respectively, than those with very high average employment income	Higher socioeconomic status was associated with increased risk of sports/recreational injury among adolescents
5	Pattussi, Hardy, & Sheiham. 2006	The odds of having dental injuries in boys decreased by 45% (OR = 0.55, 95%CI = 0.32–0.81) per unit increase in neighborhood social capital index. The relationship was not significant in girls	Higher social capital index was protective against dental injuries in boys but not girls
6	Mecredy, Janssen, & Pickett. 2012	(1) The relative odds of being injured while playing in the street was not significantly greater in children living in neighborhoods with the highest, second to highest, and third to the highest quintile of parks/recreational facilities versus those living in neighborhoods with the lowest quintile. However, the relative odds of street injury was 69% greater in children living in neighborhoods with second to the lowest quintile of parks/recreational facilities (OR = 1.69, 95%CI = 1.05–2.71) versus those living in neighborhoods with the lowest quintile	Increased number of parks and recreational facilities was not associated with increased risk of street injury while playing
		(2) The relative odds of being injured while biking/cycling in the street was more than two times greater in neighborhoods with low street connectivity (OR = 2.33, 95%CI = 1.28–4.25) versus those with high street connectivity	Lower street connectivity was associated with increased risk of biking/cycling injuries
7	Mutto, Lawoko, Ovuga, & Svanstrom. 2012	The odds of being injured was about 4 times (OR = 4.08, 95%CI = 1.12–18.67) and 7 times (OR = 6.85, 95%CI = 1.42–33.15) greater for students attending school in urban and peri-urban locations, respectively, than those attending school in rural location	Schools situated in urban and peri-urban locations increased risk of childhood and adolescent injuries when compared with those situated in rural locations
8	Gropp, Janssen & Pickett. 2012	Urban community status resulted in 1.64-fold increase in the relative odds of active transportation injury for students compared to rural community status (OR = 1.64, 95%CI = 1.14–2.36). Results of the association between neighborhood-level factors and walking/running and bicycling injuries were not reported	Living or attending schools in urban communities increased the risk of active transportation injuries for students
9	Byrnes, King, Hawe, Peters, Pickett & Davison. 2015	Lack of permanent road access lowered the risk of injury by 11% (RR = 0.89, 95%CI = 0.80–0.98)	Lack of access to road was protective against injury

Lower neighborhood income was found to be associated with reduced risk of SRI among adolescents with the odds of having SRI 20% lower in neighborhood with medium average income and 19% lower in neighborhood with high average income compared to those with very high average income (Simpson et al. 2005).

Lower street connectivity was found to be associated with increased risk of biking/cycling injuries among adolescents with the relative odds of being injured while biking/cycling in the street more than two times greater in neighborhoods with low street connectivity versus those with high street connectivity (Mecredy et al. 2012).

Among adolescents, having more parks and recreational facilities in a neighborhood was not always associated with an increased risk of street injury while playing among adolescents (Mecredy et al. 2012). For example, divide all neighborhoods into five equal groups by how many parks and recreational facilities are available. Each group includes enough neighborhoods to represent 20% of all parks and recreational facilities. We would not find a statistically significant difference in the relative odds of adolescents being injured in the street when comparing the neighborhoods with the most parks/recreational facilities (top 60% vs bottom 20%). However, if we compare only the bottom two groups of neighborhoods (those with the fewest parks), we find that adolescents living in the neighborhoods in the next to bottom group actually have 69% greater relative odds of being injured while playing in the street than adolescents in neighborhoods with the least amount of parks/recreational facilities.

Finally, it was found that living or attending schools in urban communities resulted in a 1.64-fold increase in the relative odds of active transportation injury in students compared to living in rural communities (Gropp et al. 2012).

### Discussion

Our findings suggest that more effort should be made to capture information on SRI and neighborhood characteristics when capturing data on individual-level health behaviors and outcomes. This will make it possible for more studies to examine the simultaneous effects of individual-level and neighborhood-level exposures on SRI risks. Of the nine studies reviewed, only three examined SRI as the main outcome or one of the main outcomes (Gropp et al. 2012; Mecredy et al. 2012; Simpson et al. 2005), suggesting a limited understanding of the direct and indirect role of neighborhood characteristics on SRI risk. Results from these few studies show that higher socioeconomic context (i.e., higher average employment income (Simpson et al. 2005)), lower street connectivity (Mecredy et al. 2012), and living or attending schools in urban communities (Gropp et al. 2012) were associated with increased risk of SRI after adjusting for individual-level and other neighborhood-level risk factors for SRI.

Most of the neighborhood factors associated with increased risk of SRI in the studies we reviewed are factors that have been shown to increase physical activity. For instance, a systematic review by An et al. reported that the availability of recreational facilities was positively associated with physical activity (An et al. 2019). Another systematic review found in some studies that children living in poorer neighborhoods showed lower level of physical activity compared to those living in wealthier neighborhoods (Kim et al. 2019). Lower levels of physical activity have also been reported in rural areas compared to urban areas (Martin et al. 2005). For neighborhood street connectivity, a systematic review observed in most studies reviewed that higher neighborhood street connectivity was associated with higher level of physical activity (Jia et al. 2021). This report about street connectivity and physical activity in combination with the observed relationship with SRI that we found for studies in this review suggests that higher neighborhood street connectivity can both increase physical activity and protect against SRI in children. Findings from this review provide information on neighborhood environments where proper safety precautions should be taken by participants of sports and recreational activities to protect themselves against injuries while maintaining or increasing their physical activity level. Adequate safety measures should be put in neighborhood environments, such as streets with lower connectivity and urban areas, to prevent SRI.

Results of how injuries in general are associated with neighborhood socioeconomic context are mostly different from what we found for SRI. For example, Haynes et al. (2003) found that neighborhood material deprivation increased the risk of all injuries in children presented at hospital Emergency Department in the city of Norwich, UK (Table 5). Also, Kendrick et al. (2005) and Simpson et al. (2005) reported that injury hospitalization was higher in deprived neighborhoods than in affluent neighborhoods in Nottingham, UK and in Canada, respectively (Table 5). Because SRI risks increase with increasing physical activity levels, neighborhood environments that enhance physical activity, such as affluent neighborhoods with sidewalks and bike lanes, may increase the risks of SRI while generally reducing the risks of all other injuries. The higher risk of SRI in urban areas versus rural areas could be because of the greater presence of physical activity-promoting resources, such as parks and recreational facilities, walking trails, sidewalks, bike lanes, improved street connectivity, street lighting, easy and safe street crossings, traffic calming, street beautification, mixed land use zoning, and transit-oriented development, in urban areas compared to rural areas. The higher socioeconomic status of many residents of urban areas compared to residents of rural areas could also explain the higher risks of SRI among urban residents since higher socioeconomic status is often associated with higher risks of SRI while generally lowering the risks of other injuries. Our finding supports the significance of studying the different types of injuries (e.g., SRI) separately to identify their individual relationships with neighborhood socioeconomic context. This will help in developing the right prevention intervention for the neighborhood environment associated with higher risks of each injury type.

While this study screened articles that examined people of all age groups, all nine studies that were selected for review focused on only children and adolescent population, suggesting the need for studies that assess the multilevel effects of individual and neighborhood characteristics on the risk of unintentional injuries among adult populations. Also, all the three studies that assessed the multilevel risk factors for SRI were focused only on Canadian children and adolescents with studies lacking in other developed and developing countries (Gropp et al. 2012; Mecredy et al. 2012; Simpson et al. 2005). To know if the relationships observed in Canada hold true elsewhere, other studies need to examine populations in other parts of the world.

In addition to the observed limitations related to the study population in the reviewed articles, we observed limitations in their study design. All reviewed studies used observational study design which made it difficult to determine if there was a causal relationship between neighborhood characteristics and SRI risk. Considering that experimental studies, which make it possible to determine causality in relationships, are not feasible in many instances, researchers should identify possibilities of using quasi-experimental designs because of their higher internal validity when compared to observational study designs (Ferdinand et al. 2012). Also, all three studies that focused on SRI as outcome employed cross-sectional study design, making it impossible to assess changes in SRI rates in response to changes in neighborhood environment.

The quality assessment ratings of the reviewed studies were not correlated with the quality of information reported for the multilevel models. For example, most of the reviewed studies, including those with very high-quality ratings, did not provide the necessary report to justify their use of multilevel models. Only one study reported about intraclass correlation coefficient (**ICC**) or variance partition coefficient (**VPC**) for the null model (Gropp et al. 2012), an estimate that allows for the quantification of the proportion of total variance in injuries that is attributable to neighborhood-level differences. Two other studies reported about the variance due to neighborhood-level differences for the null model with one testing for the statistical significance of the unexplained variance (Haynes et al. 2003; Mutto et al. 2012); however, they both did not report the ICC or VPC for the null model. Report on the ICC or VPC for the null model helps to assess if the need exists to use a multilevel model rather than a classical regression model (Woltman et al. 2012). Higher ICC or VPC justifies the use of multilevel models because it indicates that the proportion of total variance in injuries that is attributable to neighborhood-level differences is high and, therefore, a model that includes neighborhood-level factors to explain the existing variance is needed. Also, only two studies out of the nine studies reviewed reported about random effects for the multilevel models (Simpson et al. 2005; Kendrick et al. 2005). Another two studies tested for cross-level interaction even though reports of random effects were not provided (Pattussi et al. 2006; Sellström et al. 2003). The presence of significant random effects provides justification to test for cross-level interaction (CLI). Therefore, by not reporting or testing for random effects, these studies failed to provide a justification to test for CLI. In addition, only about half of reviewed studies reported the unexplained variance or the proportion of variance explained by the neighborhood-level factors for the final multilevel models, thereby limiting our understanding of the strength of neighborhood-level factors in explaining the variation in SRI.

Multilevel models can help us understand the direct, indirect, or interactive effects of neighborhood-level risk factors on SRI. For example, it is possible that neighborhood-built environment, a direct risk factor for SRI, can modify or moderate the relationship between individual socioeconomic status and SRI risks. However, failure to test for CLIs limits our understanding of these important relationships. To increase understanding of the multilevel determinants of injuries, there is the need for consistency in how statistical analysis is carried out and how results of studies are reported (McClure et al. 2015).

#### Limitations

The literature search strategy used in our review restricted our search results to peer review articles published in English language only. As a result, articles published in other languages and gray literature may have been excluded from our study. However, considering the significant number of peer review studies published in English language and the low number of articles that were eligible for this review, the chances of excluding additional articles published in other languages and gray literature are slim.

#### Future study directions

Future studies on the multilevel effects of neighborhood on SRI should be carried out in countries where studies are currently missing such as the USA and low- and medium-income countries (**LMIC**). While capturing contextual level information might be a challenge in many LMIC, this is not the case in the USA and many other developed countries since this information has been captured in many primary and secondary data studies for other health outcomes and behaviors (e.g., physical activity and obesity) (Rundle et al. 2007, 2009; Sallis et al. 2009). The reason for the lack of studies in the USA, for example, is likely due to the fact that many primary and secondary data capturing information on health behaviors and outcomes do not capture information on SRI and those that capture information on individual SRI risk often fail to capture contextual level information. This mean that researchers who wants to assess the association between neighborhood context and SRI might be required to apply more complex methods such as data linkage and analysis of nested or hierarchical data. For researcher who need to collect primary data, contextual level information can be collected by self-administered questionnaires or telephone interviews, by neighborhood audits, and by GIS-based measures that are derived from existing data sources with spatial reference (Brownson et al. 2010).

Only two of the studies we reviewed were carried out in LMIC (Pattussi et al. 2006; Mutto et al. 2012), indicating that more future studies should investigate the effect of neighborhood on SRI in LMIC and do comparative analyses of the relationships in LMIC versus developed countries. Future studies should also investigate how other neighborhood variables that enhance walkability or bikeability are associated with SRI. Two of the reviewed studies that examined neighborhood effect on SRI assessed the relationship between neighborhood street connectivity and SRI (Gropp et al. 2012; Mecredy et al. 2012). While street network connectivity may be an indicator that a neighborhood is walkable or bikeable, other neighborhood variables, such as the presence and quality of sidewalks and bike lanes or a composite score of the density of neighborhood attributes of interest, diversity of land use, street design, and accessibility to destination of interest, may be better indicators of neighborhood walkability (Freeman et al. 2013). Also, the effects of historic and present-day neighborhood segregation and social vulnerability [factors that weaken a neighborhood’s ability to respond to hazardous events such as injury (CDC/ATSDR SVI Fact Sheet|Place and Health|ATSDR 2021)] on SRI should be examined as these factors have been found to be associated with other health outcomes and health behaviors including physical inactivity and obesity (Nardone et al. 2020a, 2020b; Krieger et al. 2020; An and Xiang 2015).

## Conclusion

This review systematically analyzed studies that applied multilevel models to assess the effects of neighborhood-level risk factors on SRI. Only nine studies met our eligibility criteria for inclusion in this review and among them only three examined SRI as the main outcome or one of the main outcomes. These studies showed that neighborhood-level factors, such as higher socioeconomic context, lower street connectivity, and living or attending schools in urban communities, were associated with increased risk of SRI. While these findings provide evidence that neighborhood-level factors in addition to individual-level factors should be taken into consideration when developing public health policies for injury prevention, more multilevel studies should be carried to strengthen this evidence in order to better inform SRI prevention policy decisions. Four quality assessment criteria out of 15 were met by all nine studies including clearly specifying and defining the study population, selecting study subjects from similar population at same time period, pre-specifying and uniformly applying inclusion and exclusion criteria for all study participants, and adjusting for key confounding variables. However, only a few studies provided report of study participation rate of ≥ 50% for eligible persons (*n* = 4), measured exposures of interest prior to measuring study outcomes (*n* = 2), and had sufficient timeline to increase the probability of finding significant association between exposure and outcome if it existed (*n* = 2). None of the studies used experimental or quasi-experimental design. Future studies should identify possibilities of using experimental or quasi-experimental designs so that they can easily determine if there is a causal relationship between neighborhood characteristics and SRI risk. Also, longitudinal studies should be explored so that changes in SRI rates in response to changes in neighborhood environment can be assessed. Future studies should also provide a more coherent report of the results of multilevel models, one that presents estimates that help to (1) justify the use of multilevel models, (2) justify the test for cross-level interactions when examined, (3) determine the strength of neighborhood-level factors in explaining the variation in SRI which will help to provide a better understanding of the impact of neighborhood characteristics on SRI risk.

## Data Availability

All information generated or analyzed during this systematic review are included in this published article.

## Abbreviations

SRI: Sports and recreational Injury
ED: Emergency department
TBI: Traumatic brain injury
MLM: Multilevel model
CLI: Cross-level interaction
PRISMA: Preferred Reporting Items for Systematic reviews and Meta-Analyses
PROSERO: International Prospective Register of Systematic Reviews
NIH: National Institute of Health
ICC: Intraclass correlation coefficient
VPC: Variance partition coefficient
CD: Cannot determine
NA: Not applicable
NR: Not reported
